# Comprehensive Dipeptide Analysis Revealed Cancer-Specific Profile in the Liver of Patients with Hepatocellular Carcinoma and Hepatitis

**DOI:** 10.3390/metabo10110442

**Published:** 2020-11-01

**Authors:** Hitoshi Ozawa, Akiyoshi Hirayama, Futaba Shoji, Midori Maruyama, Kumi Suzuki, Hisami Yamanaka-Okumura, Hiroshi Tatano, Yuji Morine, Tomoyoshi Soga, Mitsuo Shimada, Masaru Tomita

**Affiliations:** 1 Institute for Advanced Biosciences, Keio University, Tsuruoka, Yamagata 997-0052, Japan; 038hts@sfc.keio.ac.jp (H.O.); dofla20mingo@yahoo.co.jp (F.S.); green12@ttck.keio.ac.jp (M.M.); ksuzuki@ttck.keio.ac.jp (K.S.); soga@sfc.keio.ac.jp (T.S.); mt@sfc.keio.ac.jp (M.T.); 2 Systems Biology Program, Graduate School of Media and Governance, Keio University, Fujisawa, Kanagawa 252-0882, Japan; 3 Department of Clinical Nutrition and Food Management, Graduate School of Biomedical Sciences, Tokushima University Graduate School, Tokushima 770-8503, Japan; okumurah@tokushima-u.ac.jp (H.Y.-O.); h-tatano@u-shimane.ac.jp (H.T.); 4 Department of Health and Nutrition, Faculty of Nursing and Nutrition, The University of Shimane, Izumo, Shimane 693-8550, Japan; 5 Department of Digestive and Pediatric Surgery, Graduate School of Medical Sciences, Tokushima University, Tokushima 770-8503, Japan; ymorine@tokushima-u.ac.jp (Y.M.); mitsuo.shimada@tokushima-u.ac.jp (M.S.)

**Keywords:** dipeptide, hepatocellular carcinoma, hepatitis B virus, hepatitis C virus, liquid chromatography tandem mass spectrometry, capillary electrophoresis tandem mass spectrometry, metabolomics

## Abstract

As the physical properties and functionality of dipeptides differ from those of amino acids, they have attracted attention in metabolomics; however, their functions in vivo have not been clarified in detail. Hepatocellular carcinoma (HCC) is the most common type of primary liver cancer, and its major cause is chronic hepatitis. This study was conducted to explore tumor-specific dipeptide characteristics by performing comprehensive dipeptide analysis in the tumor and surrounding nontumor tissue of patients with HCC. Dipeptides were analyzed by liquid chromatography tandem mass spectrometry and capillary electrophoresis tandem mass spectrometry. Principal component analysis using 236 detected dipeptides showed differences in the dipeptide profiles between nontumor and tumor tissues; however, no clear difference was observed in etiological comparison. In addition, the N- and C-terminal amino acid compositions of the detected dipeptides significantly differed, suggesting the substrate specificity of enzyme proteins, such as peptidase. Furthermore, hepatitis-derived HCC may show a characteristic dipeptide profile even before tumor formation. These results provide insight into HCC pathogenesis and may help identify novel biomarkers for diagnosis.

## 1. Introduction

Hepatocellular carcinoma (HCC) is the third leading cause of cancer-related deaths worldwide [1]. A major cause of HCC is chronic hepatitis caused by hepatitis virus infection. Infection with hepatitis B virus (HBV) or C virus (HCV) causes hepatitis, and long-term destruction and regeneration of hepatocytes leads to cirrhosis and, finally, HCC [2]. The most characteristic feature of HCC is that tumor growth is rapid and initial symptoms are unlikely to be detected. When a tumor is found, it has often spread to other organs [3].

An important approach to understanding the characteristics of liver cancer is metabolome analysis, which can reveal the metabolite profile in vivo. Metabolome analysis has been applied to various tumor tissues such as gastric cancer [4], liver cancer [5,6], prostate cancer [7,8], breast cancer [9], oral cancer [10], and lung cancer [7,11]. Additionally, several studies have reported the discovery of potential serum biomarkers of HCC by metabolome analysis using gas chromatography-mass spectrometry (GC-MS) and liquid chromatography-mass spectrometry (LC-MS) [12,13,14,15].

As post-amino acids, dipeptides are highly diverse with different physical and functional properties from amino acids [16]. In recent years, dipeptides have attracted attention as functional biomaterials, particularly as potential disease biomarkers [17]. For instance, carnosine and anserine containing an imidazole group derived from histidine can remove reactive oxygen and thus play a role as endogenous antioxidants [18,19]. In addition, leucine-histidine suppresses microglia activity, reduces proinflammatory cytokine production, and ameliorates depression and depression-related emotional disturbances [20]. It has also been reported that dipeptides consisting of aromatic amino acids and leucine, such as Tyr-Leu, Phe-Leu, and Trp-Leu, have anxiolytic-like activity in mice [21]. The physiological activity of artificially synthesized dipeptides has also been reported [22,23].

Some dipeptides have also been used as biomarkers of disease. For example, prolyl-4-hydroxyproline, a dipeptide produced when collagen is degraded, is used as a urinary biomarker of bone resorption [24,25]. Furthermore, we found that the concentration of γ-glutamyl dipeptides in the serum fluctuates in nine types of liver diseases such as hepatitis, cirrhosis, and HCC, indicating the potential of these biomarkers for liver disease screening [26,27].

Thus, analyzing dipeptides in a biological sample may lead to the discovery of new functional components and various disease biomarkers. Although some dipeptides have been measured, few comprehensive dipeptide analyses have been performed. One reason for this is that all dipeptides, except those composed of the same amino acid, have structural isomers with opposite amino acid binding orders. As these isomers have the same molecular weight, it is difficult to distinguish them by mass spectrometry. Therefore, these isomers must be separated by chromatography before being introduced into a mass spectrometer, but it is difficult to separate many dipeptides by a single analytical method. To overcome this limitation, we recently developed a comprehensive dipeptide analytical method using liquid chromatography tandem mass spectrometry (LC-MS/MS) and capillary electrophoresis tandem mass spectrometry (CE-MS/MS), which enabled simultaneous quantitation of 335 types of dipeptides [16].

In this study, we applied LC-MS/MS and CE-MS/MS to compare the dipeptide profiles of tumors and surrounding nontumor tissues of patients with liver cancer. The characteristics of the amino acids constituting the dipeptide detected in the tissues were also examined. Furthermore, the dipeptide profiles in HCC with different etiologies were compared. It was found that the dipeptide profiles in non-tumor and tumor tissues differed, and hepatitis-derived cancer has a characteristic dipeptide profile before tumor onset.

## 2. Results

### 2.1. Study Population and Data Analysis

Tumor and surrounding nontumor liver tissues were surgically resected from 13 patients with HCC and 3 patients with metastatic liver cancer. The clinical information of the patients is listed in Table 1. All liver cancer cases were the first instance of cancer, and the patients had no treatment history prior to surgery. The mean ± standard deviation of the patients’ age and body mass index were 67.6 ± 9.3 years and 22.6 ± 3.6, respectively. The HCC group contained 6 non-B/C samples, 2 HBV samples, and 5 HCV samples. The stages of cancer in HCC varied from I to IVB.

In this study, 140 and 96 dipeptides were detected in the liver by LC-MS/MS and CE-MS/MS, respectively (quantitative results are shown in Supplementary Table S1). Among the total of 236 dipeptides detected in both methods, the peak of 14 dipeptides could not be distinguished by MRM transition and retention time. The amount of each dipeptide was standardized to nmol/g liver tissue, and subsequent analysis was performed using this value. Additionally, the amino acids comprising the dipeptides were expressed as one-letter codes.

### 2.2. Outlier Analysis

**Figure 1:**
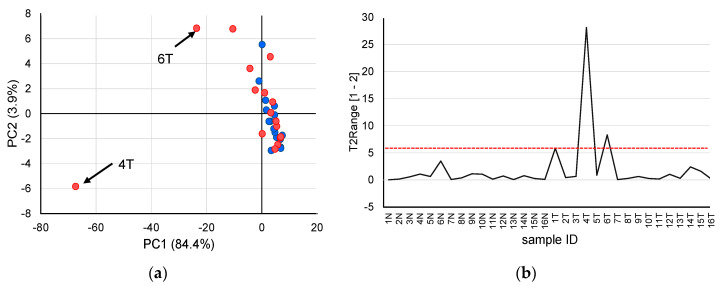
Outlier analysis based on quantified dipeptides obtained from patients with liver cancer. (**a**) principal component analysis score plots using the auto-scaled dipeptide data of paired non-tumor (blue) and tumor (red) tissues. The contribution ratios were 84.4% and 3.9% for PC1 and PC2, respectively. (**b**) Hotelling’s T^2^ range plot of all samples. The red dashed line indicates the upper control limit (*α* = 0.01).

First, principal component analysis (PCA) was performed to identify trends in the dipeptide profiles of all samples measured in this study (Figure 1). The results of the PCA score plots (Figure 1a) suggested that two tumor tissues were supposed outliers. Therefore, outlier analysis was carried out to investigate whether outliers were included in the measured samples. Figure 1b shows the results of outlier analysis using Hotelling’s T^2^ statistics in PCA. As Hotelling’s T^2^ statistics of two tumor tissues (No. 4T and 6T) exceeded the upper control limit at a significance level of 0.01, these samples were considered outliers and excluded from subsequent analysis.

### 2.3. Principal Component Analysis

**Figure 2:**
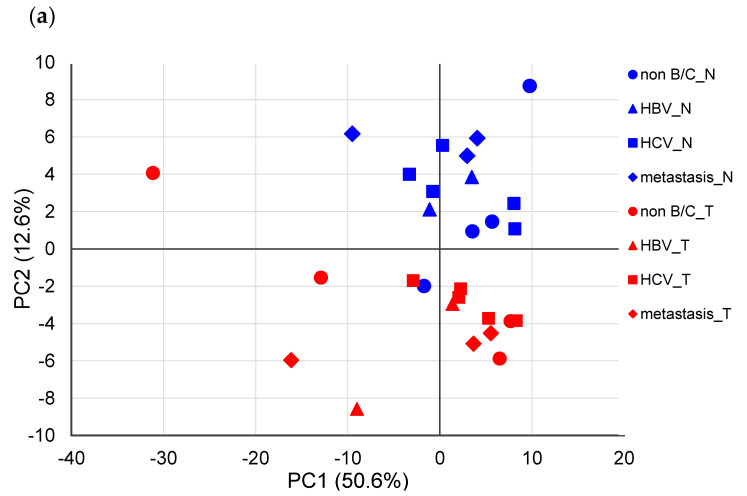

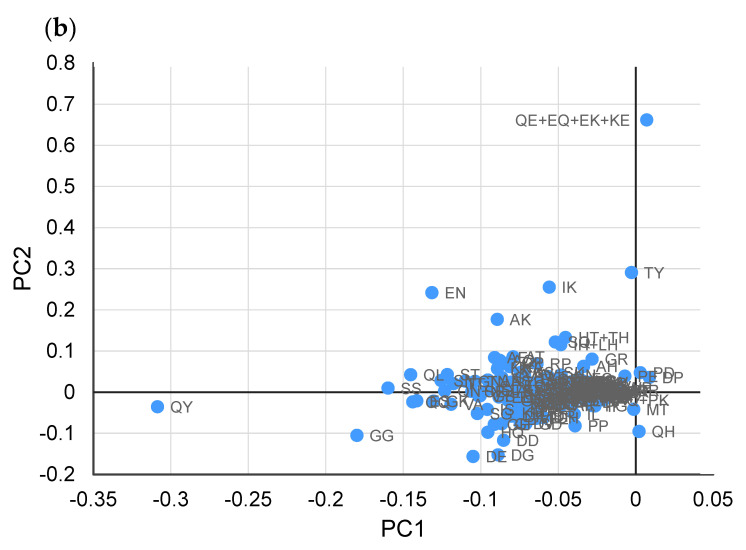
Principal component analysis (PCA) using the Pareto-scaled dipeptide data. (**a**) PCA score plots of paired nontumor (blue) and tumor (red) tissues in different types of liver cancers. The contribution ratios were 50.6% and 12.6% for PC1 and PC2, respectively. (**b**) PCA loading plots of dipeptides on the first two principal components.

Next, PCA based on Pareto scaling was performed using the remaining 14 samples (each sample contained non-tumor and tumor tissues, respectively), excluding samples showing outliers (Figure 2). In the PCA score plot (Figure 2a), nontumor and tumor tissues were sufficiently separated mainly by principal component 2. In addition, QE (Gln-Glu) + EQ (Glu-Gln) + EK (Glu-Lys) + KE (Lys-Glu) (overlapped peak) and TY (The-Tyr), IK (Ile-Lys), and EN (Glu-Asn) showed relatively large values in principal component 2 of the loading plot (Figure 2b), suggesting that these dipeptides contributed to the separation of nontumor and tumor tissues.

### 2.4. Characteristics of Dipeptides Detected in Liver Tissue

**Figure 3:**
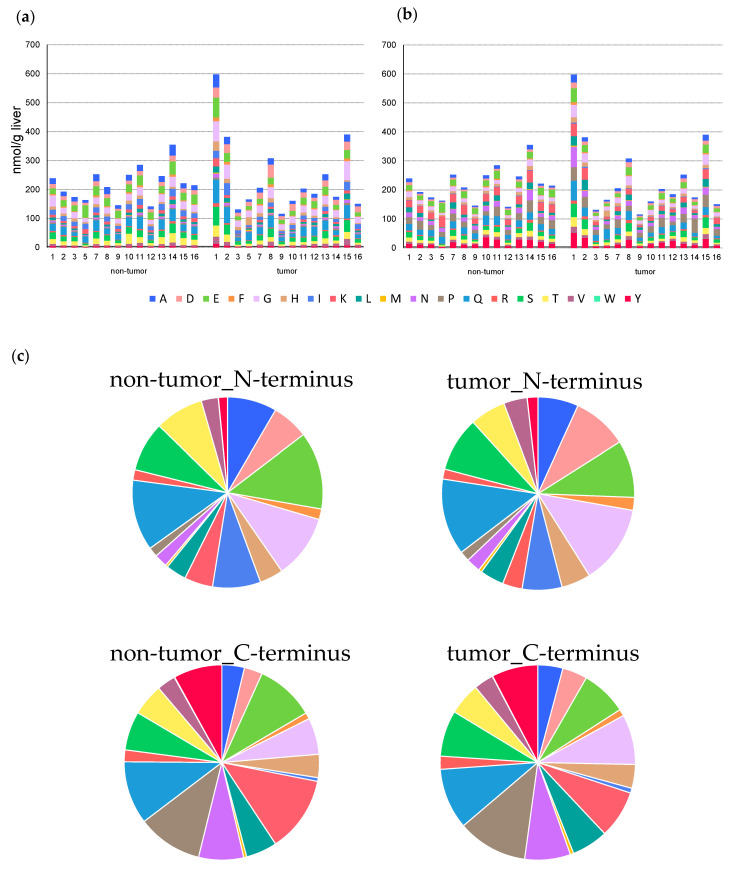
Amino acid composition of N-terminus (**a**) and C-terminus (**b**) in dipeptide detected from each liver tissue. The columns represent the number of moles (nmol/g liver). (**c**) The difference in the average composition ratio of N-terminal and C-terminal amino acids in each tissue.

To determine the characteristics of the dipeptides detected in each sample, grouping was performed for each amino acid constituting the N-terminus (Figure 3a) and C-terminus (Figure 3b). A relatively high accumulation of dipeptide was observed in the nontumor tissue of sample ID14 and tumor tissues of samples ID1, 2, and 15, but no obvious difference was observed in the amino acid composition of these samples compared to the other samples. This trend was similar between nontumor and tumor tissues. The amino acid compositions at the C- and N-termini significantly differed (Figure 3c). For example, dipeptides containing alanine (A), aspartic acid (D), and isoleucine (I) were predominant at the N-terminus, whereas dipeptides containing lysine (K), asparagine (N), proline (P), and tyrosine (Y) were increased at the C-terminus.

### 2.5. Changes in Dipeptide Profile with and Without Hepatitis in HCC

**Figure 4:**
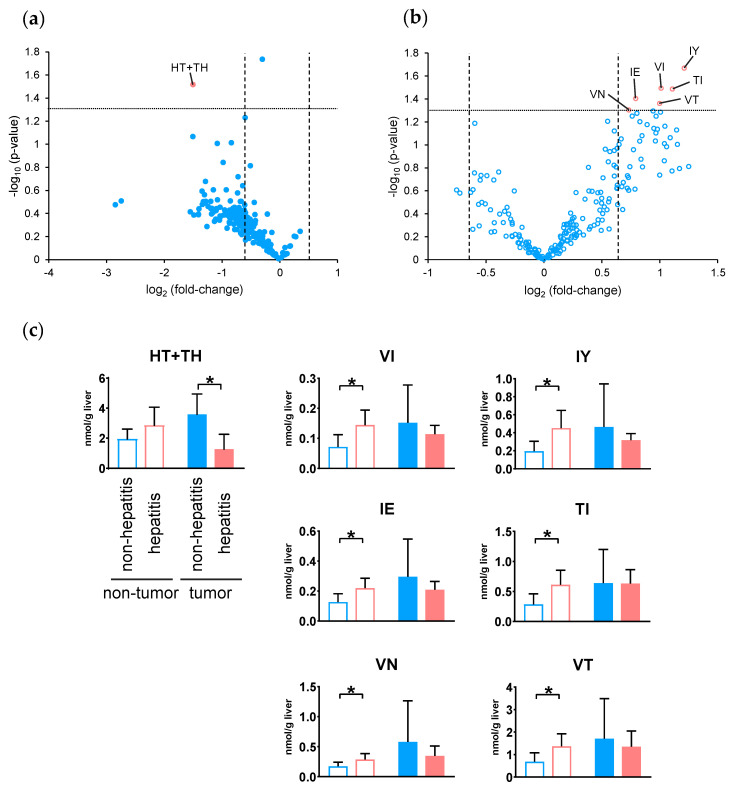
Volcano plot of differential dipeptides in the tumor (**a**) and nontumor (**b**) tissues with and without hepatitis in hepatocellular carcinoma. For each dipeptide, the −log_10_ (*p*-value) was plotted vs. log_2_ (fold-change). (**c**) Dipeptides showing a significant difference in the volcano plot (*p* < 0.05 and fold-change < 0.67 or > 1.5). The columns represent the average number of moles (nmol/g tissue), and error bars indicate the standard deviation. * *p* < 0.05.

The dipeptide profiles of non-hepatitis- and hepatitis-derived HCC were compared. Volcano plots were prepared using the quantitated values of dipeptides detected in non-hepatitis (non B/C, *n* = 4) and hepatitis (HBV or HCV, *n* = 7) samples from the tumor (Figure 4a) and nontumor tissues (Figure 4b) of HCC, respectively. In tumor tissues, only the amount of HT + TH was significantly decreased (*p* < 0.05, fold-change < 0.67) in hepatitis samples compared to non-hepatitis samples. Additionally, a significant increase (*p* < 0.05, fold-change > 1.5) in 6 dipeptides in hepatitis samples, including VI, IY, IE, TI, VN, and VT, was observed in the nontumor tissues. According to the results of the volcano plot, the number of dipeptides that was increased in hepatitis samples was slightly higher in nontumor tissues, whereas this number was mostly decreased in tumor tissues.

Finally, we investigated the relationship of the dipeptide abundance between non-tumor and tumor tissues with and without hepatitis. Figure 4c shows the number of dipeptides in both tissues, showing significant differences in either tissue. One of the dipeptides that showed significance in tumor tissue showed no significance in nontumor tissue, whereas six of the dipeptides that showed significance in nontumor tissue showed no significance in tumor tissue.

## 3. Discussion

In this study, we performed a comprehensive dipeptide analysis of paired tumors and surrounding nontumor liver tissues obtained from 13 patients with HCC and three patients with metastatic liver cancer. We successfully quantified 236 dipeptides (14 overlapped) with our previously developed method using LC-MS/MS and CE-MS/MS [16]. This study reports the largest number of dipeptides detected in the liver tissue to date.

Unlike the analysis of body fluids such as blood and urine, it is necessary to consider variability between samples when performing metabolome analysis of tissues. Particularly, tumor tissues exhibit cancer heterogeneity, and the amounts of metabolites and dipeptides may greatly differ depending on the sample location. Therefore, effectively removing outliers is useful for searching for effective biomarkers in subsequent analyses. In this study, PCA using auto-scaled dipeptide data revealed that the score plots of samples 4T and 6T tended to deviate from the score plots of the other samples. Therefore, in outlier analysis with Hotelling’s T^2^ statistics, these two samples exceeded the upper control limit at a significance level of 0.01 and were therefore excluded from subsequent analysis.

PCA was performed again on the remaining 14 nontumor and tumor tissues using Pareto-scaled dipeptide data, which showed that nontumor and tumor tissues were separated for principal component 2. In contrast, no significant difference was observed depending on the factors causing liver cancer. These results demonstrate that the difference in the dipeptide profile in the liver depends on whether it is a nontumor- or tumor-derived tissue and does not depend on the factors causing cancer. This finding is similar to the serum metabolic profiles obtained by LC-MS from two HCC cohorts infected with HBV or HCV [28].

The characteristics of the detected dipeptides were also examined. A comparison of the total amount of dipeptides in nontumor and tumor tissues in each specimen showed that 7 of 14 specimens showed an increase, and the others showed a decrease in the amounts of dipeptides in the tumor tissue. When amino acids in various tumor tissues were measured by metabolome analysis [7,29], the samples showed different tendencies, possibly because of the heterogeneity of cancer. In addition, no significant difference was found in the amino acid composition between nontumor and tumor tissues, whereas a significant difference was observed at the N- and C-termini. One possible cause for this is the substrate specificity of the peptidase. For example, chymotrypsin, a serine protease, specifically cleaves the C-terminal of aromatic amino acids. In addition, elastase, which has a shallow bottom in the substrate-binding pocket, specifically cleaves the peptide bond at the C-terminus of amino acids with small side chains. However, various factors require further analysis, such as where the dipeptides detected in the liver tissue are produced.

Although there are several routes to HCC development, HBV and HCV infections are the most important risk factors [30]. Therefore, investigating the differences in dipeptide profiles depending on the presence or absence of hepatitis is important for understanding the process of HCC development. In addition, determining the differences in dipeptide profiles will enable identifying the biomarker candidates for future evaluation using biofluids. Nevertheless, there are limited numbers of studies searching for biomarkers by measuring dipeptides in body fluids. Recently, the dipeptides, such as hydroxyproline-Leu, EW, and FF, have been selected as promising predictive biomarkers for the diagnosis of epithelial ovarian cancer [31].

In this study, analysis of dipeptides with and without hepatitis revealed that seven dipeptides were significantly changed (*p* < 0.05, fold-change < 0.67 or >1.5). Among the tumor tissues, HT + TH was significantly decreased in hepatitis samples, and most other dipeptides also tended to decrease. In contrast, in nontumor tissues, VI, IY, IE, TI, VN, and VT were significantly increased in hepatitis samples, and many other dipeptides also tended to increase. For the seven dipeptides showing significant differences, the amounts in corresponding tissues were also examined, but none of the dipeptides showed a common change between tissues. Thus, hepatitis-derived HCC showed a characteristic tendency, with the amount of dipeptide increased in the surrounding nontumor tissue but decreased in the tumor tissue. This suggests that the change in the dipeptide profile due to the presence or absence of hepatitis already occurred before tumor formation and was maintained throughout the production of different dipeptides and metabolic mechanisms even after tumor generation.

There were several limitations to this study. First, because the number of samples used in this study was relatively small, the power of statistical analysis may not have been sufficient. In addition, as only liver cancer was analyzed, it is necessary to investigate other liver diseases such as cirrhosis and nonalcoholic steatohepatitis. Further studies of larger sample sizes are needed to examine the roles of dipeptides in liver cancer in detail.

In conclusion, we detected 236 dipeptides in liver cancer tissue using a comprehensive dipeptide analytical method involving CE-MS/MS and LC-MS/MS. Similar to previously reported metabolite results, the dipeptide profiles in nontumor and tumor tissues differed, although no clear difference was observed in the etiological comparison. We also found that the N- and C-terminal amino acid compositions of the detected dipeptides significantly differed in both tissues, suggesting the substrate specificity of enzyme proteins, such as peptidase. A comparison of the dipeptide profiles depending on the presence or absence of hepatitis suggested that hepatitis-derived cancer has a characteristic dipeptide profile before tumor onset.

## 4. Materials and Methods

### 4.1. Sample Collection and Dipeptide Extraction

This study was approved by the Tokushima University Hospital Ethics Committee (Approved no. 1815), and the corresponding regulatory agencies and all experiments were carried out in accordance with approved guidelines. All patients involved in the study signed an informed consent form.

Tumor and surrounding nontumor tissues were surgically resected from 13 patients with HCC and 3 patients with metastatic liver cancer. The resected tissue samples were quickly frozen at −80 °C until sample preparation.

Liver tissues were weighed (~100 mg) and homogenized in methanol (500 μL) containing internal standards (20 μM methionine sulfone and 50 μM Phe-Gly-^13^C_9_-^15^N_1_) using a Shake Master NEO instrument (Biomedical Science Co., Ltd., Tokyo, Japan). The homogenates were mixed with chloroform (500 μL) and Milli-Q water (200 μL), and the mixture was centrifuged at 4600× *g* for 15 min at 4 °C. The upper aqueous layer (300 μL) was centrifugally filtered through a 5-kDa cutoff filter (Human Metabolome Technologies, Tsuruoka, Japan) to remove proteins. The filtrate was centrifugally concentrated in a vacuum evaporator and dissolved in Milli-Q water (25 μL) immediately before CE-MS/MS analysis. This sample was diluted by 5-fold with Milli-Q water and subjected to LC-MS/MS analysis.

### 4.2. Dipeptide Analysis

Comprehensive dipeptide analysis was performed by CE-MS/MS and LC-MS/MS, as described previously [16]. The CE-MS/MS system was composed of an Agilent 1600 CE system (Agilent Technologies, Santa Clara, CA, USA), Agilent 6490 triple quadrupole mass spectrometer, Agilent 1200 series isocratic HPLC pump, and Agilent G1607A CE-ESI-MS sprayer kit. The separation was carried out on a fused-silica capillary (50 μm I.D., 360 μm O.D., 135 cm total length, Polymicro Technologies, Phoenix, AZ, USA) using 200 mM of aqueous acetic acid as a background electrolyte. Prior to the first use, a new capillary was rinsed with background electrolyte for 20 min. Equilibration was performed for 4 min by flushing with background electrolyte before each run. The sample solution was injected at 5 kPa for 15 s (~15 nL), and a positive voltage of 30 kV was applied. The capillary was maintained at 20 °C, and the sheath liquid (methanol: water = 1:1, *v*/*v*) was delivered at 10 μL/min.

ESI-MS/MS analysis was conducted in positive ion mode using the following source parameters: dry gas temperature, 280 °C; dry gas flow rate, 11 L/min; nebulizer pressure, 10 psi; capillary voltage, 4 kV; fragmentor voltage, 380 V; cell accelerator voltage, 7 V; high- and low-pressure radiofrequency voltage of ion funnel, 150 and 60 V, respectively; and dwell time, 5 ms.

LC-MS/MS analysis was carried out using an Agilent 1290 Infinity HPLC system coupled to an Agilent 6490 triple quadrupole mass spectrometer with an Agilent jet stream ESI interface. Dipeptides were separated on an Acquity UPLC HSS PFP column (2.1 × 150 mm, 1.8 μm; Waters, Milford, MA, USA). The mobile phase was composed of 0.1 vol% aqueous formic acid (A) and 0.1 vol% formic acid in 95 vol% aqueous acetonitrile (B). The flow rate was 0.2 mL/min, and the following linear gradient was used: 0–3 min, 1% B; 3–30 min, 1% to 50% B; 30–30.1 min, 50% to 99% B; 30.1–35 min, 99% B; 35–35.1 min, 99% to 1% B, followed by equilibration with 1% B for 15 min. The injection volume was 1 μL, and the column temperature was maintained at 45 °C.

Agilent jet stream-ESI-MS/MS analysis was performed in positive ion mode using the following source parameters: dry gas temperature, 280 °C; dry gas flow rate, 12 L/min; nebulizer pressure, 30 psi; sheath gas temperature, 380 °C; sheath gas flow rate, 12 L/min; capillary voltage, 3.5 kV; nebulizer voltage, 2.0 kV; fragmentor voltage, 380 V; cell accelerator voltage, 7 V; high- and low-pressure radiofrequency voltage of the ion funnel, 150 and 60 V, respectively; and dwell time, 2 ms.

The molar amount of each dipeptide in 1 g of liver tissue was determined by normalizing the peak area of each dipeptide with respect to the area of the internal standard and by using the single-point standard calibration curves (CE-MS/MS; 20 μM, LC-MS/MS; 15 μM).

### 4.3. Outlier Analysis

To examine the outliers, the *T*^2^ statistic and upper control limit were used to evaluate the results of PCA with autoscaling [32]. The *T*^2^ statistic in each sample was evaluated using Equation (1).

(1) $$T_{i}{}^{2}=M_{i}{}^{2}=X_{ci}P_{A}L^{-1}P_{A}{}^{t}X_{ci}{}^{t}$$

where *M_i_* is the Mahalanobis distance, *X_ci_* is the line vector of the standardized data of dipeptide amount, *P_A_* is the matrix including the eigenvector of principal component *A*, and *L* is the diagonal matrix of the eigenvalue of principal component *A*.

The control limit was evaluated using Equation (2).

(2) $${\mathrm{CL}}_{1-\alpha }=\frac{{\left(n-1\right)}^{2}}{n}x_{1-\alpha }^{\beta }\left[\frac{A}{2},\frac{n-A-1}{2}\right]$$

where *n* is the number of specimens, *A* is the number of principal components, α is the significance level (*α* = 0.01), and $x_{1-\alpha }^{\beta }$ is the (1−α)-quantile of the beta distribution of parameter [A/2,(n−A−1)/2].

### 4.4. Data Analysis

The acquired data were analyzed using the MassHunter software (version B.06.00, Agilent Technologies). PCA was performed using the SIMCA software (version 13, Umetrics AB, Umeå, Sweden). The Mann-Whitney U-test was used for statistical analysis. A *p*-value of less than 0.05 was considered statistically significant.

## Figures and Tables

**Table 1:** Clinical characteristics of patients in this study.

ID	Age	Sex	BMI	Type	Virus	Stage
1	68	Female	31.9	HCC	Non B/C	III
2	73	Female	23.1	HCC	Non B/C	II
3	73	Male	17.3	HCC	Non B/C	II
4	72	Male	24.7	HCC	Non B/C	II
5	63	Male	18.2	HCC	Non B/C	II
6	67	Male	- *	HCC	Non B/C	III
7	44	Male	22.3	HCC	HBV	II
8	65	Male	19.0	HCC	HBV	III
9	61	Male	21.8	HCC	HCV	II
10	57	Male	21.5	HCC	HCV	I
11	78	Female	20.5	HCC	HCV	III
12	75	Male	25.4	HCC	HCV	II
13	60	Male	21.8	HCC	HCV	IVB
14	78	Female	22.1	MLC	Non B/C	
15	68	Female	23.3	MLC	Non B/C	
16	80	Male	26.7	MLC	Non B/C	

BMI, body mass index; HCC, hepatocellular carcinoma; MLC, metastatic liver cancer; Non B/C, Non B-Non C hepatitis; HBV, hepatitis B virus; HCV, hepatitis C virus. *, missing value.

## Supplementary Materials

The following are available online at https://www.mdpi.com/2218-1989/10/11/442/s1, Table S1: Amount of dipeptide quantified in non-tumor and tumor tissues of patients with HCC and MLC (nmol/g liver).
